# Corticospinal Tract Development, Evolution, and Skilled Movements

**DOI:** 10.1002/mds.30199

**Published:** 2025-04-25

**Authors:** Emmanuel Roze, Caroline Dubacq, Quentin Welniarz

**Affiliations:** ^1^ Sorbonne Université, INSERM, CNRS, Paris Brain Institute Institut du Cerveau Paris France; ^2^ Département de Neurologie Assistance Publique – Hôpitaux de Paris, Hôpital Pitié‐Salpêtrière Paris France

**Keywords:** axon guidance, cortico‐motoneuronal connection, mammalian evolution, motor system, voluntary movement

## Abstract

The evolution of the corticospinal tract (CST) is closely linked to the development of skilled voluntary movements in mammals. The main evolutionary divergence concerns the position of the CST within the spinal cord white matter and its postsynaptic targets in the grey matter. Here, we examine the developmental steps contributing to the CST projection pattern from an evolutionary point of view. Recent studies have highlighted the molecular mechanisms involved in these processes and how they relate to the acquisition of skilled movements. Comparison of the evolution of the CST in different species offers a new perspective on manual dexterity. In particular, it adds a new level of complexity to the classic view linking the evolution of the CST and the sequential improvement of skilled hand movements from rodents to primates. © 2025 The Author(s). *Movement Disorders* published by Wiley Periodicals LLC on behalf of International Parkinson and Movement Disorder Society.

## Introduction

The corticospinal tract (CST) is the primary motor pathway responsible for voluntary motor control. Its evolution is closely linked to the development of skilled voluntary movements in mammals. Part of the anatomical organization of the CST is common to all mammalian species. Most CST axons originate from neurons located in the inferior part of the cortical layer V in the primary motor cortex. Other regions, such as the sensory cortex, contribute to the CST to a lesser extent. After leaving the neocortex, the CST runs through the internal capsule and mesencephalic cerebral peduncle before reaching the pons and medulla oblongata in a ventral position. In most mammals, the CST crosses the midline at the junction between the brainstem and the spinal cord, the “pyramidal decussation”, before entering the contralateral spinal cord. CST axons exit the spinal cord white matter at specific levels to reach their targets in the grey matter. The main evolutionary divergence concerns the position of the CST within the spinal cord white matter and its projection pattern in the grey matter, which has switched from a dorsal to a ventral position from mice to humans.^1^ This anatomical divergence paralleled the emergence of direct cortico‐motoneuronal connections in higher primates resulting in increased manual dexterity.^2^

The establishment of the CST projection pattern implies various developmental steps that we previously reviewed extensively.^1^ Here, we provide an update by examining the chronological sequence of CST development and focusing on: (1) the lateralization of the CST through axonal guidance at the midline; (2) the determination of the CST target along the rostrocaudal axis of the central nervous system (CNS); (3) the connection of the CST axons with their spinal targets; and (4) the refinement of the CST projections. Recent studies comparing CST development and anatomy across different species have identified their underlying molecular mechanisms and how they relate to skilled movements. These studies add a new level of complexity to the classic view linking the evolution of the CST and the sequential improvement of skilled hand movements from rodents to primates.

## Lateralization of CST Projections

### CST Guidance at the Midline and Lateralization of Motor Control

**FIG. 1:**
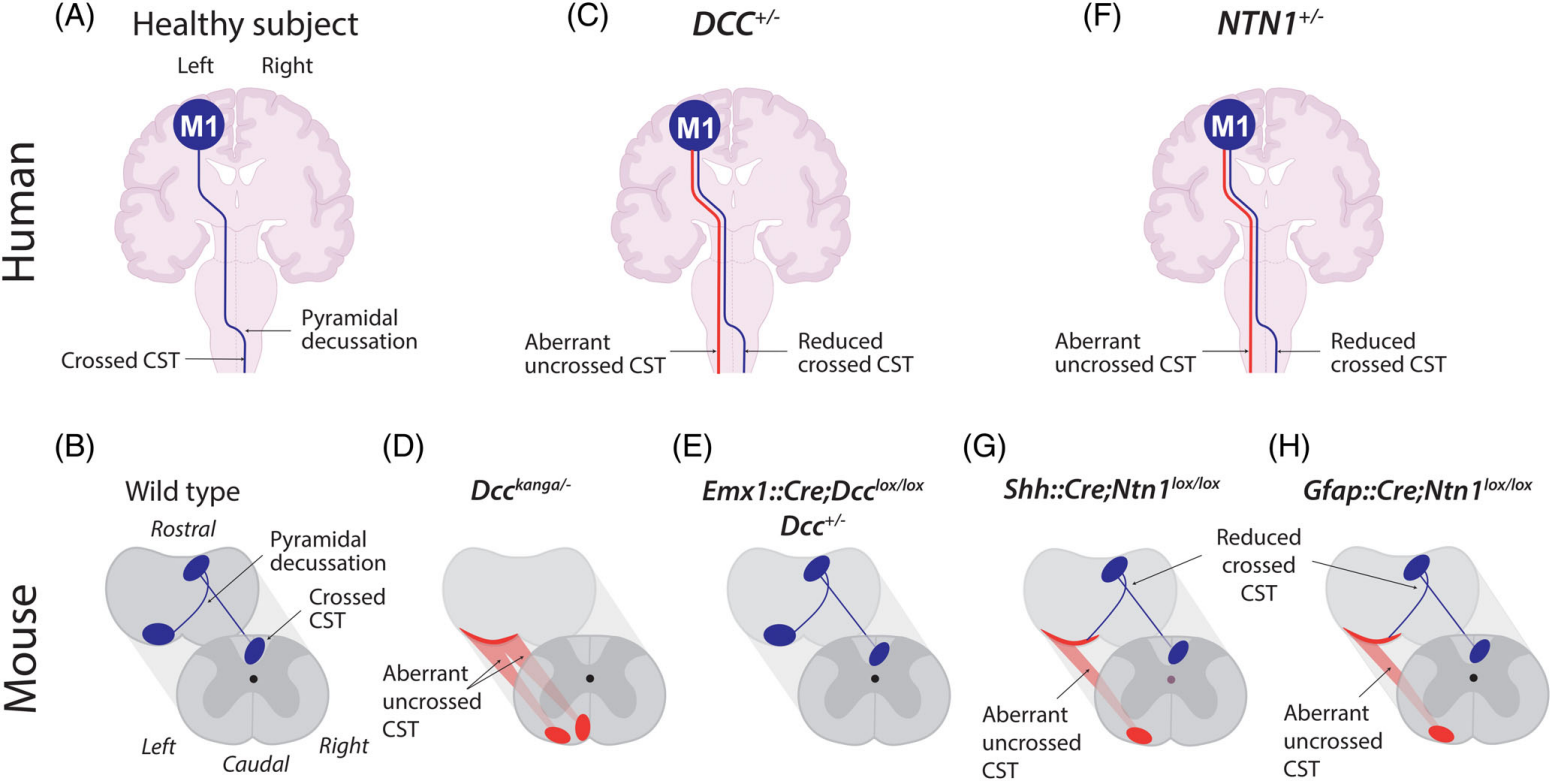
Corticospinal tract anatomy and lateralization of motor control in humans and mice. (A) Schematic representation of the left corticospinal tract within the human central nervous system (CNS). The corticospinal tract (CST) axons originate in the primary motor cortex (M1) and cross the midline at the brainstem/spinal cord junction. (B) Schematic representation of the left corticospinal tract within the mouse CNS at the brainstem (left) and spinal cord (right) levels. The CST axons cross the midline at the brainstem/spinal cord junction. (C) In *DCC* ^*+/−*^ patients, transcranial magnetic stimulation (TMS) and magnetic resonance imaging (MRI) tractography suggest abnormal guidance of the CST at the pyramidal decussation resulting in bilateral projections to the spinal cord. (D) In *Dcc* ^*kanga*^ mice, the CST fails to cross the midline at the pyramidal decussation, and remains in the ventral funiculus of the ipsilateral spinal cord. (E) Anatomy of the CST is normal when *Dcc* is deleted in the neocortex and thus in the CST in *Emx1::Cre;Dcc* ^*lox/lox*^ conditional mice or in heterozygous *Dcc* ^*+/−*^ mice. (F) In *NTN1* ^*+/−*^ patients, TMS and MRI tractography suggest abnormal guidance of the CST at the pyramidal decussation resulting in bilateral projections to the spinal cord. Netrin‐1 suppression in the floor plate (G) or in the ventricular zone (H) in *Shh::Cre;Dcc* ^*lox/lo*^, or *Gfap::Cre;Dcc* ^*lox/lox*^ conditional mice results in a similar phenotype: most CST axons fail to cross the midline and remain in the ventral ipsilateral spinal cord, while a small proportion crosses the midline at the pyramidal decussation. Dark blue: normal anatomy of the CST. Red: aberrant anatomy of the CST. [Color figure can be viewed at wileyonlinelibrary.com]

Lateralization of motor control refers to the ability to produce purposeful asymmetric movements.^3^ In mammals, lateralized movements of the upper limbs underlie a vast repertoire of behaviors, from food handling in mice to complex bimanual skills in humans such as playing the piano. The exact role of the CST in motor output production remains a subject of ongoing debate and likely varies across mammalian species. In species with direct cortico‐motoneuronal projections, the prevailing model posits that the CST conveys motor commands to motoneurons, though alternative models have also been proposed.^4^ In rodents which lack direct cortico‐motoneuronal projections, the CST might have limited influence on motoneurons, and could instead modulate spinal sensory circuits.^5^, ^6^ Nevertheless, the CST seems to play a conserved role in the lateralization of motor control across mammals.^3^, ^7^ In most mammals, the CST controlling distal extremities crosses the midline at the brainstem/spinal cord junction, the “pyramidal decussation”, and projects to the spinal cord contralateral to the hemisphere of origin (Fig. 1A,B). In mice and humans, abnormal guidance of the CST at the pyramidal decussation resulting in bilateral – instead of contralateral – projections to the spinal cord (Fig. 1C–H) is associated with mirror movements (MMs).^1^ These are involuntary movements on one side of the body that mirror voluntary movements on the other side and constitute the main neurological manifestation in human congenital mirror movement disorder (CMM) (Panel 1). CMM thus provide a unique model to study lateralization of motor control. To date, the genes associated with CMM are: *DCC*, *NTN1*, *RAD51*, *DNAL4*, and more recently *ARHGEF7*.^8^, ^9^, ^10^, ^11^, ^12^, ^13^, ^14^ In the next section, we will focus on the roles of *DCC/Dcc* and *NTN1/Ntn1* in the development of the CST, which have been extensively studied in humans and mice.

### Role of DCC */*Netrin‐1 in CST Guidance at the Midline and Lateralization of Motor Control in Mammals

Netrin‐1, the product of the *NTN1* gene, is an extracellular protein that acts as a guidance cue for many commissural axons in the CNS, in particular through its receptor DCC.^15^, ^16^, ^17^ Mice completely lacking *Dcc* or *Ntn1* die at birth^18^, ^19^ when CST axons reach the pyramidal decussation. The study of mouse models and human patients with a partial defect has shed a new light on the role of these genes in the formation of the pyramidal decussation and MMs.

*Dcc* ^*kanga*^ mice carrying a defective but viable biallelic *Dcc* variant completely lack the pyramidal decussation (Fig. 1D)^20^, ^21^ and present a “hopping gait” phenotype: they produce symmetric instead of asymmetric limb movements during stereotypic locomotion. Conditional deletion of *Dcc* in the spinal cord reproduces this phenotype,^22^ suggesting that the hopping gait is not the equivalent of human MMs but rather corresponds to a dysfunction of the spinal circuitry.^3^ *Dcc* ^*kanga*^ mice also produce increased voluntary symmetric forelimb movements during exploratory reaching behavior.^21^ This phenotype is reminiscent of MMs and has been associated with an abnormal CST laterality projection pattern.^3^ In *Emx1::Cre;Dcc* ^*lox/lox*^ mice, DCC is selectively suppressed in forebrain cells expressing Emx1 (including CST neurons) by the use of the Cre‐lox system.^23^ The CST trajectory is unaffected in these mice.^21^ Thus, CST midline crossing is impaired in complete *Dcc* ^*kanga*^ mutants, while specific deletion of *Dcc* in the CST does not alter the pyramidal decussation (Fig. 1E).^21^ This demonstrates that the role of *Dcc* in CST guidance at the midline is indirect, as it does not require *Dcc* expression in the CST.

In patients with monoallelic *DCC* mutations,^8^, ^12^, ^24^ single‐pulse transcranial magnetic stimulation over the cortical hand representation results in bilateral instead of strictly contralateral motor evoked potentials in hand muscles with identical latencies.^21^ This reflects the existence of fast‐conducting CST projections emerging from one hemisphere to both sides of the spinal cord. Diffusion tensor imaging has revealed abnormal CST midline crossing at the pyramidal decussation, with an increased proportion of uncrossed CST fibers (Fig. 1C).^21^, ^25^ By contrast, mice with monoallelic *Dcc* mutations have a normal pyramidal decussation. Although these mice have subtle motor deficits, lateralization of motor control is preserved, suggesting the involvement of compensatory mechanisms.^21^, ^26^ Altogether, *DCC/Dcc* mutations thus result in abnormal guidance of the CST at the pyramidal decussation and in reduced ability to produce asymmetric voluntary movements in both humans and mice.

*Shh::Cre;Ntn1* ^*lox/lox*^ mice with a conditional deletion of *Ntn1* in the floor plate of the caudal brainstem and spinal cord also have an abnormal pyramidal decussation.^27^ Some CST fibers manage to turn dorsally and cross the midline, but most CST axons remain on the ventral funiculus of the ipsilateral spinal cord, resulting in bilateral CST projections (Fig. 1G). These mice produce a higher proportion of symmetric movements during exploratory reaching and adaptive locomotion, two tasks involving a descending cortical control.^7^, ^28^, ^29^, ^30^, ^31^ In addition, it has recently been shown that the absence of netrin‐1 in the ventricular zone of the hindbrain using a *Gfap::Cre* conditional mouse line leads to a similar but milder phenotype (Fig. 1H).^32^ Thus, both floor plate‐ and ventricular zone‐derived netrin‐1 proteins are important for CST guidance at the pyramidal decussation. Accordingly, electrophysiological and magnetic resonance imaging (MRI) data indicate that patients with a monoallelic *NTN1* mutation have an abnormal pyramidal decussation with bilateral CST projections to the spinal cord (Fig. 1F).^10^ In vitro experiments suggest that the level of netrin‐1 in the extracellular compartment is reduced in these patients. In keeping with this, a recent study identified *ARHGEF7*, a downstream effector of the netrin‐1/DCC complex, as a new CMM gene.^13^

Taken together, these results show that the role of DCC and netrin‐1 in CST guidance at the pyramidal decussation and lateralization of motor control is conserved in mammals from mice to humans.

## CST Projection Pattern along the CNS Rostrocaudal Axis

### Molecular Control of CST Projections Along the Spinal Rostrocaudal Axis

**FIG. 2:**
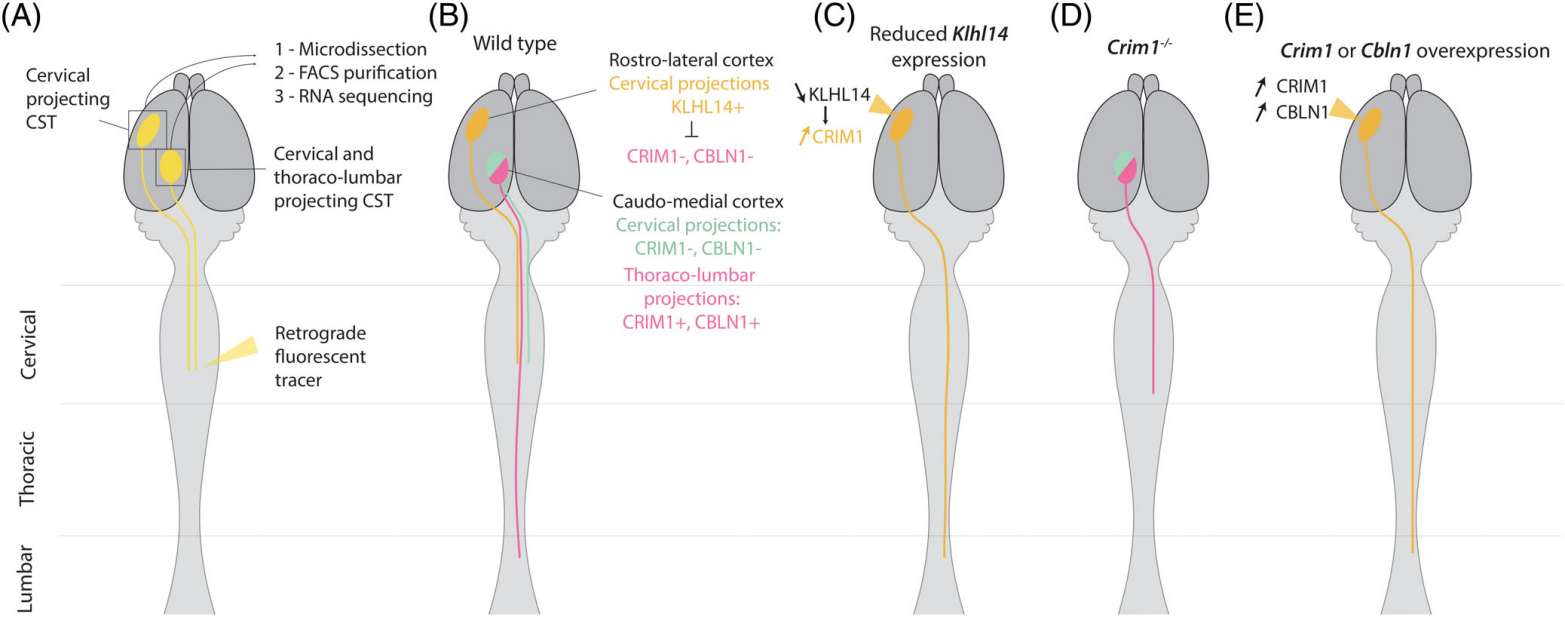
Molecular control of corticospinal tract (CST) projections along the rostrocaudal axis of the central nervous system in mice. (A) Retrograde injection of a fluorescent tracer into the cervical spinal cord at different developmental stages combined with microdissection of the cortex, fluorescence‐activated cell sorting (FACS) purification, and RNA sequencing led to the identification of distinct genes involved in the specification of cervical and thoracolumbar projecting CST populations. (B) In the rostrolateral cortex, *Klhl14* expression represses *Crim1* and *Cbln1* expression and drives CST projections toward the cervical spinal cord. In the caudomedial cortex, CRIM1 and CBLN1 specify a subpopulation of CST neurons projecting to the thoracolumbar spinal cord. (C) KLHL14 reduction or conditional absence in the rostrolateral cortex results in ectopic *Crim1* expression and redirects CST projections of these neurons toward the thoracolumbar spinal cord. (D) *Crim1* deletion results in a lack of CST neurons projecting beyond the cervical spinal cord. (E) Overexpression of *Crim1* or *Cbln1* in the rostrolateral cortex redirects CST axons toward the thoracolumbar spinal cord. Yellow: retrograde fluorescent tracer. Orange: rostrolateral CST. Green: caudomedial CST projecting to the cervical spinal cord. Pink: caudomedial CST projecting to the thoracolumbar spinal cord. [Color figure can be viewed at wileyonlinelibrary.com]

A fundamental level of organization in the motor system involves distinct corticospinal circuits targeting specific segments of the spinal cord. CST neurons driving forelimbs and hindlimbs movements target the cervical and lumbar spinal cord, respectively. The cortical somatotopic arrangement of CST neurons differs across mammalian species. In rodents, the cortical distribution of forelimbs and hindlimbs CST neurons partially overlaps.^33^, ^34^ This contrasts with the primate's motor cortex where cervical and lumbar projecting CST populations are clearly segregated.^35^ Interestingly, in macaque monkey, the somatotopic organization of CST fibers is no longer present in the spinal cord, where the axons of forelimb and hindlimb CST neurons are intermingled.^36^ Similar results were found in mice.^37^ This implies that the targeting of specific spinal segments by different CST populations is not regulated by spatial segregation in the brainstem and spinal cord, but rather relies on genetic mechanisms. In rodents, the molecular mechanisms translating the somatotopic organization of the motor cortex into the specification of distinct CST populations with specific targets along the rostrocaudal axis have recently been elucidated.^37^, ^38^, ^39^, ^40^ In mice, CST neurons of the rostrolateral cortex homogeneously project to the cervical spinal cord. By contrast, CST neurons of the caudomedial sensorimotor cortex have more heterogeneous targets as cervical and thoracolumbar projecting CST neurons are intermingled. Mouse CST populations projecting to the cervical and thoracolumbar spinal cord, respectively, have been isolated using a combination of retrograde CST tracing with microdissection of the motor cortex and fluorescence‐activated cell sorting (FACS) purification at early developmental stages (Panel 2, Fig. 2A). Transcriptional analyses identified several genes defining distinct CST populations with specific segmental targets.^37^ *Crim1* and *Klhl14* are critical players for the development of CST thoracolumbar and cervical projections, respectively. *Klhl14* is expressed by CST neurons in the rostrolateral cortex projecting to the cervical spinal cord. Conversely, *Crim1* is expressed by CST neurons in the caudomedial sensorimotor cortex projecting to the thoracolumbar spinal cord (Fig. 2B). The expression of *Crim1* during early development, even before CST neurons have reached the spinal cord, predicts thoracolumbar projections at maturity. Critically, this molecular pattern is not a mere reflection of the spatial segregation of cervical and thoracolumbar projecting neurons in the cortex. Indeed, within the caudomedial sensorimotor cortex, cervical and thoracolumbar projecting CST neurons are spatially intermingled but present distinct gene expression patterns. Thoracolumbar projecting CST neurons express *Crim1*, while cervical projecting CST neurons do not (Fig. 2B). Reduced expression of KLHL14 in the lateral cortex results in *Crim1* ectopic expression and aberrant CST extension into the thoracolumbar spinal cord (Fig. 2C).^38^ In mice with a deletion of *Crim1*, CST axons do not extend past the upper spinal thoracic level (Fig. 2D). *Crim1* misexpression in the lateral cortex, which normally gives rises to cervical projections, redirects CST axons toward thoracolumbar targets (Fig. 2E). The authors further identified *Cbln1* (Cerebellin 1) as another gene sufficient, though not required, for CST axon extension to the thoracolumbar spinal cord (Fig. 2E).^39^ CST axons from the lateral cortex that aberrantly reach the thoracolumbar segments in *Crim1* and *Cbln1* overexpression experiments fail to extend collaterals in the spinal cord grey matter. This suggests that distinct molecular pathways underlie CST axon extension within the white matter of the spinal cord and axon collateralization into the grey matter.

Using similar methods in mice (Panel 2), a subsequent study investigated the molecular factors distinguishing motor cortex projection neurons (which target the distal brainstem and spinal cord) from visual cortex neurons, which project to more proximal areas such as thalamus and pons.^41^ This study identified three transcription factors highly specific to motor cortex projection neurons (ZBTB16, MEIS2, and NFIA). Downregulation of these factors in distally projecting motor cortex neurons led to a redirection of their axon toward proximal targets, such as the thalamus.

Among the molecular determinants of CST neurons, small leucine‐rich proteoglycan (SLRP) components of the extracellular matrix such as lumican, are specifically expressed by rostrolateral CST neurons in both mouse and marmoset brains.^42^ In the mouse, lumican is secreted by rostrolateral CST axons in the cervical spinal cord. Its suppression results in increased innervation of cervical segments by caudomedial CST neurons and reduced skilled forelimb reaching movements. This indicates that lumican inhibits collateralization of caudomedial CST neurons in the cervical spinal cord in a non‐cell‐autonomous manner.^42^ Rostrolateral CST neurons are evolutionarily newer in mice. This inter‐axonal competition monitored by lumican could have promoted the integration of an evolutionarily newer CST population (rostrolateral) in a pre‐existing circuit by inhibiting cervical innervation from evolutionarily older populations (caudomedial). This competition for the innervation of the cervical spinal cord between molecularly distinct CST populations might have contributed to the refinement of forelimb motor skills across mammals.

Physical factors such as axon bundle fasciculation also proved to be important in establishing specific CST connections along the rostrocaudal axis. Deletion of the plexin receptors plexin‐A1 and plexin‐A3 or their ligands semaphorin‐5A and semaphorin‐5B can cause CST defasciculation in the spinal cord starting at the decussation level.^43^ As a result, hindlimb CST neurons, which normally reach the lumbar spinal segment, exit the white matter of the spinal cord at the medulla level and aberrantly innervate the cervical spinal cord instead.

### CST Collateral Projection Pattern along the CNS Rostrocaudal Axis

CST axons terminate their trajectory in the spinal cord. However, CST collaterals do not only reach spinal segments, but also various additional targets along the CNS. Using viral intersectional tools (Panel 3), two recent studies mapped the projectome of CST collaterals in mice,^44^ rats, and rhesus monkeys^45^ thereby quantifying the relative distribution of CST collaterals along the rostrocaudal axis of the CNS.

Surprisingly, more than half (55%) of the collaterals from the CST axons targeting the caudal cervical spinal cord reach supraspinal targets in rats: the sensory and motor cortices, striatum, thalamus, and brainstem. In mice, supraspinal CST collaterals are mainly found in the striatum.^44^ Within the rat spinal cord, most CST terminals are located in the rostral cervical segment (28% of the entire CST terminals), while the caudal cervical segment (the final target of the labeled CST neurons) represents only 14% of total CST terminals. In rhesus macaques, most of the collaterals of CST neurons target the spinal cord (almost 70% of all CST terminals), and the proportion of supratentorial targets is far below that observed in rats (7% vs. 35%). Spinal cord terminals are mainly located in the caudal cervical segment (35% of the total CST terminals).

Overall, the distribution of CST terminals along the rostrocaudal axis of the CNS has shifted during evolution. The CST exhibits broad connections with supraspinal and spinal structures in rodents, while it is more focused on spinal motor structures in rhesus macaques.^45^

## CST Projection Pattern and Postsynaptic Targets in the Spinal Cord

### CST Projection Pattern Across Mammalian Evolution

**FIG. 3:**
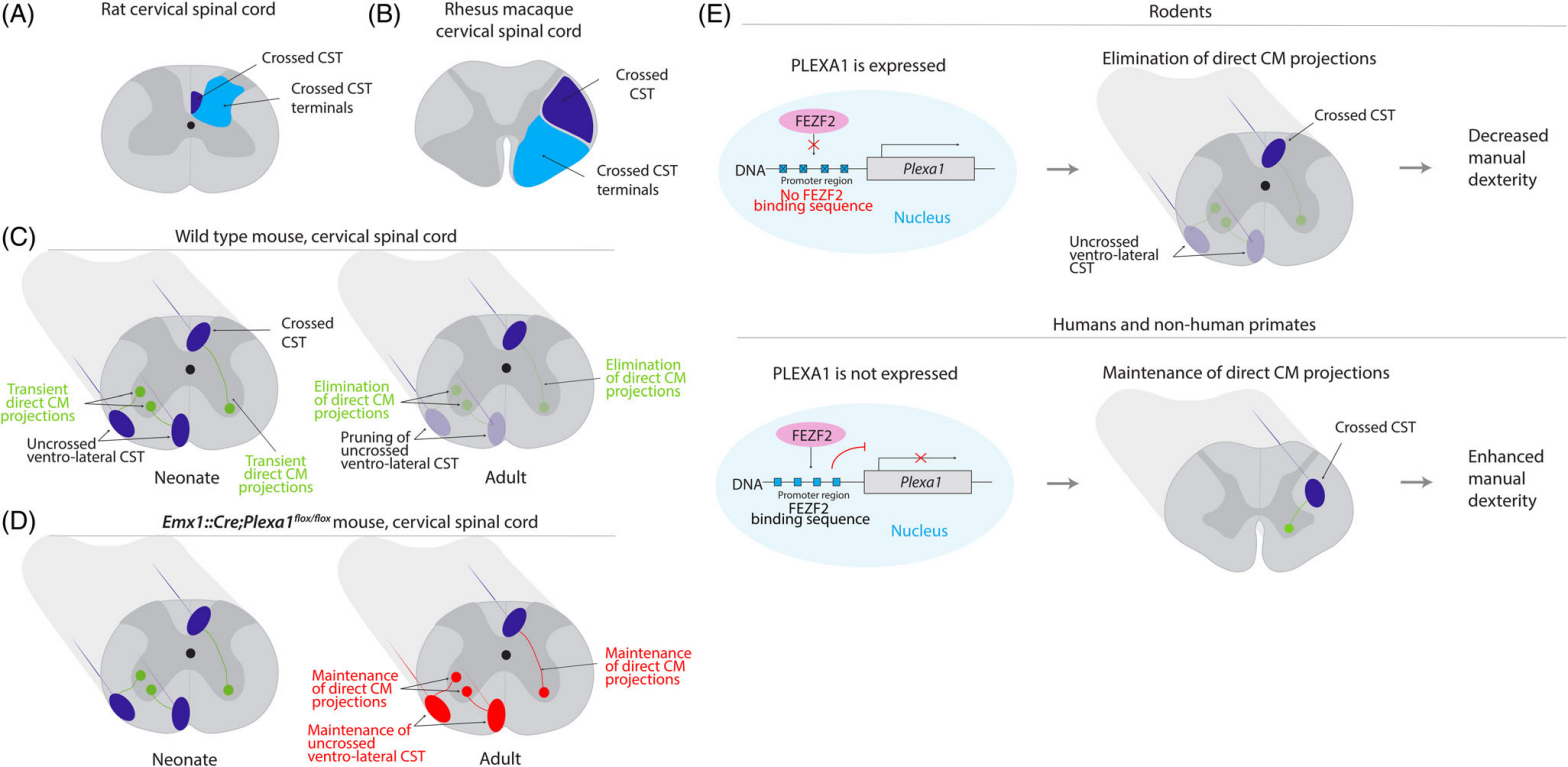
Evolution of the corticospinal tract (CST) projection pattern in the spinal cord grey matter. (A) In rats, the CST originating from the forelimb area of the motor cortex mainly projects to the intermediate and dorsal horns of the spinal cord grey matter. (B) In rhesus macaques, the CST from the hand area of the primary motor cortex mainly projects to the intermediate and ventral spinal cord. (C) In neonate mice, a contingent of uncrossed CST fibers is present in the ventrolateral spinal cord. Both the crossed dorsal and the uncrossed ventrolateral CST fibers establish transient direct cortico‐motoneuronal (CM) connections. The uncrossed ventrolateral CST and direct CM connections are eliminated during postnatal development. (D) Conditional deletion of *Plexa1* in the CST neurons results in the maintenance of direct CM connections in adult *Emx1::Cre;Plexa1* ^*floxf/flox*^ mice. (E) In rodents, FEZF2 does not bind the enhancer region of *Plexa1*. This results in *Plexa1* expression, elimination of direct CM connections, and reduced manual dexterity. In humans and non‐human primates, the evolution of *PLEXA1* promoter sequences results in FEZF2 binding, repression of *PLEXA1* expression, maintenance of direct CM connections in adulthood, and enhanced manual dexterity. Dark blue: CST. Light blue: terminations of the crossed CST. Green: direct CM connections. Red: aberrant maintenance of uncrossed CST and direct CM projection in adult mice. [Color figure can be viewed at wileyonlinelibrary.com]

The main evolutionary divergence across mammals concerns the CST position within the spinal cord white matter and its projection pattern in the grey matter. In rodents, the crossed CST originating from the forelimb area of the motor cortex runs through the dorsal funiculus and its terminations are mainly found in the dorsal and intermediate horns of the spinal cord grey matter, while terminals in the ventral horn are sparse (Fig. 3A).^46^ By contrast, in non‐human primates, the crossed CST fibers from the hand area of the primary motor cortex are located in the lateral funiculus and their terminals are located in the intermediate and ventral horns, and in particular in lamina IX containing motoneurons (Fig. 3B).^47^, ^48^ The elegant study comparing the projectome of adult rats and rhesus monkeys cited earlier also directly compared CST projections in the spinal cord grey matter using transsynaptic tracers.^45^ In rhesus monkeys, 81% of the transsynaptic targets of CST neurons were alpha‐motoneurons located in the ventral spinal cord. The remaining 19% were interneurons located in the intermediate and ventral spinal cord. This contrasts with the fact that most CST terminals are located in the intermediate spinal grey matter, in accordance with other studies.^47^, ^48^ This suggests that CST terminals in the intermediate zone could synapse onto dendrites of alpha motoneurons extending to the intermediate grey matter.^45^ Alternatively, it could indicate that a single CST fiber terminating in lamina IX contacts several motoneurons.^49^ In rats, CST neurons from the primary motor cortex mainly project to interneurons located in the dorsal grey matter. However, CST projections differ between the upper and lower cervical segments. In CST neurons controlling distal forelimb muscles, the proportion of ventral transsynaptic labeling is increased in the lower cervical segment (9%) as compared with the upper cervical segment (2%). Consistently, CST neurons mainly target motor networks in the lower cervical segments: V0c neurons, V2a motor interneurons, and indirectly alpha motoneurons. The same CST neurons extend collaterals to a distinct set of sensory interneurons in the upper cervical spinal cord.^45^ In addition, CST neurons have differential spinal targets depending on their origin in the mouse motor or sensory cortices.^50^ Motor CST neurons connect mainly with motor interneurons located in the intermediate and ventral grey matter, in particular CHX10‐positive V2a interneurons. Conversely, sensory CST neurons mainly project to VGLUT3‐positive interneurons located in the dorsal grey matter. Mice with lesions of either of these two circuits have impaired skilled reaching. Lastly, distinct CST populations within the cortical forelimb area project to distinct populations of spinal interneurons.^51^

These results are in line with the long‐time hypothesis that direct cortico‐motoneuronal connections exist only in higher primates.^2^ However, recent evidence indicates that direct cortico‐motoneuronal connections are transiently formed at early developmental stages in rodents.^52^, ^53^, ^54^, ^55^ Indeed, retrograde monosynaptic tracing from forelimb muscles injected with rabies virus at early postnatal stages results in bilateral cortical labeling in mice.^52^ Likewise, anterograde tracing of the CST during the first postnatal week reveals the presence of CST fibers not only in the contralateral dorsal funiculus, but also in the ipsilateral ventral and lateral funiculi (ventrolateral CST). Both the crossed and uncrossed CST neurons establish direct contacts with motoneurons (Fig. 3C). These direct cortico‐motoneuronal connections are no longer present after 3 weeks, suggesting that they are eliminated during the postnatal refinement of the motor circuits (Fig. 3C).^52^, ^54^, ^55^ This is in stark contrast to the situation in non‐human primates, where direct cortico‐motoneuronal projections are absent at birth and gradually increase in density throughout postnatal development.^56^

### Molecular Control of CST Projection Pattern Across Evolution

Plexin receptors and their ligands, semaphorins, play important roles during development.^57^ Plexin‐A1 and Sema6D are involved in axon guidance in the spinal cord^58^ and at the optic chiasm.^59^ Plexin‐A3 and plexin‐A4 are important for the formation of the pyramidal decussation.^60^ Deletion of the plexin‐A1 receptor in the neocortex of *Emx1::Cre;Plexa1* ^*flox/flox*^ mice results in an increased number of functional direct cortico‐motoneuronal connections in juvenile and adult mice, along with abnormal maintenance of the ipsilateral ventrolateral CST (Fig. 3D). The pyramidal decussation is unaffected in these mice.^52^ Mice lacking plexin‐A1 exhibit improved motor performances in the capellini handling test and in reaching tasks as compared with control mice, although their success rates remain far below those observed in non‐human primates.^61^ The relative contributions of the increased number of direct cortico‐motoneuronal connections and the preservation of the ventrolateral CST to the enhanced manual dexterity in these mutant mice is unclear. The *PLEXA1* gene encoding the plexin‐A1 receptor is present in humans but shows a very weak expression at week 20 post‐conception. FEZF2, a zinc‐finger transcription factor known to be involved in corticospinal neuron specification,^62^ strongly binds to the promoter region of the *PLEXA1* gene in humans but not *Plexa1* in mice. Consistently, FEZF2 represses the expression of the human but not mouse *PLEXA1/Plexa1* gene (Fig. 3E). Phylogenetic analyses revealed that functional FEZF2 binding sites in the promoter region of *PLEXA1* are present exclusively in primate species demonstrating direct cortico‐motoneuronal connections in adults. To sum up, the evolution of the *PLEXA1/Plexa1* promoter from mice to humans has resulted in increased binding of the transcription factor FEZF2, decreased expression of *PLEXA1*, and eventually maintenance of direct cortico‐motoneuronal connections in adults and increased manual dexterity (Fig. 3E). While the genetic modifications leading to the evolution of the human cortical circuitry have been extensively studied, the underlying mechanisms remain poorly understood.^63^ How can the elimination of cortico‐motoneuronal connections in rodents, rabbits, and primates such as marmosets be interpreted? Direct cortico‐motoneuronal connections and increased hand dexterity might not confer fitness advantages to quadrupedal animals, which rely more on locomotion for survival. Maintenance of direct cortico‐motoneuronal connections in adult rodents could also impair the development of other descending motor tracks.^52^, ^64^ However, these different hypotheses are not supported by direct experimental evidence. These findings challenge the classic view that direct cortico‐motoneuronal connections developed in a sequential manner during mammalian evolution.

## Refinement of the CST Projection Pattern

### Refinement of CST Projection Pattern Across Mammalian Evolution

A hallmark of neuronal development is that brain circuits initially exhibit broad connections that are later refined. The CST projection pattern to the spinal cord is refined during postnatal development in mice and humans. In both species, the CST establishes bilateral projections to the spinal cord at early developmental stages. Most of the ipsilateral projections are eliminated during postnatal development.^1^ As previously mentioned, the CST in rodents initially projects to the ipsilateral ventrolateral spinal cord, and these projections are later eliminated.^52^, ^55^ Intriguingly, transient ipsilateral CST projections have not been found during postnatal development in macaques.^56^ A recent study investigated the embryonic development of the CST in *Macaca fascicularis*.^65^ Unilateral injection of a retrograde tracer in the cervical spinal cord resulted in bilateral cortical labeling, therefore demonstrating the existence of transient ipsilateral CST projections in non‐human primates at prenatal stages. It remains unclear why ipsilateral CST projections are eliminated postnatally in rodents and humans and during embryonic development in non‐human primates.

### Molecular Control of the Refinement of the CST Projection Pattern

**FIG. 4:**
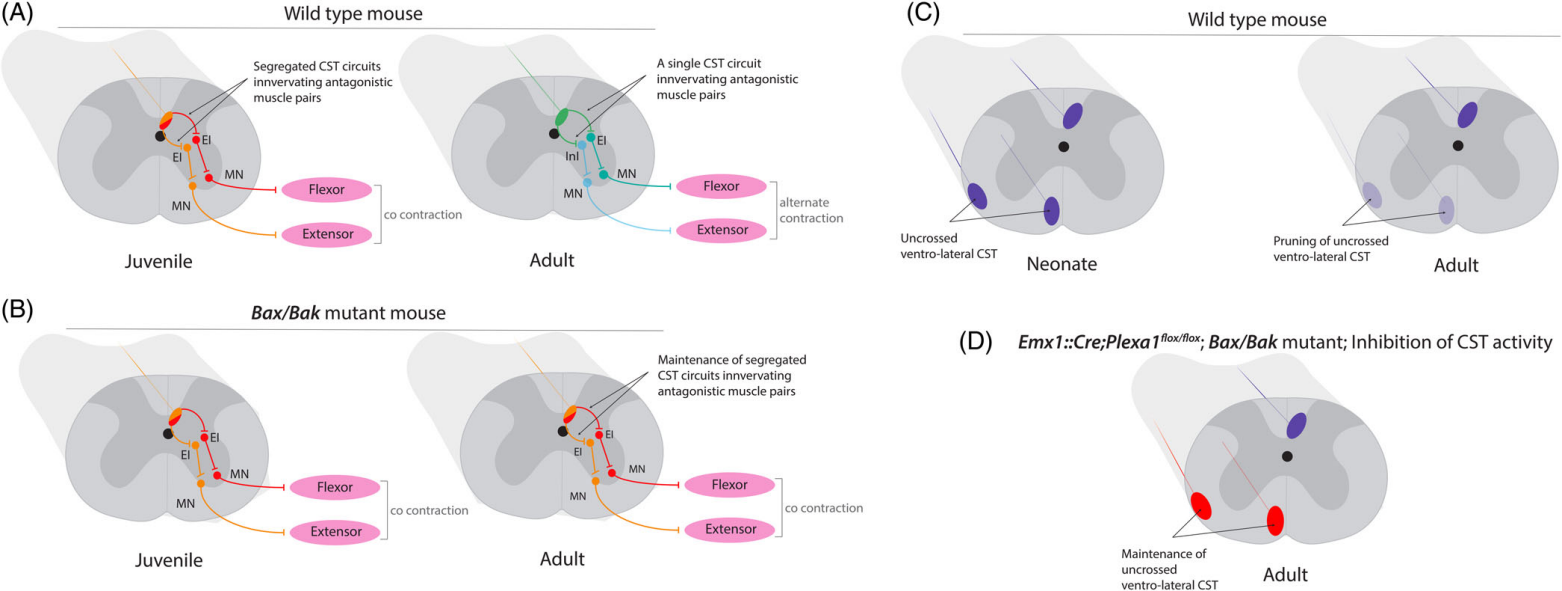
Molecular control of corticospinal tract (CST) maturation during postnatal development. (A) In juvenile mice, antagonistic muscles (for instance, flexor and extensor) are innervated by segregated CST populations. This results in concomitant activation of antagonistic muscles upon electric stimulation of the motor cortex. In adult mice, the same CST circuit innervates antagonistic muscle pairs. This results in alternate contraction of antagonistic muscles upon stimulation of the motor cortex. (B) The refinement of antagonistic muscle pairs circuits is impaired in *Bax/Bak* double mutants. (C) In neonate mice, an uncrossed contingent of CST fibers is present in the ventrolateral spinal cord. These fibers are eliminated during postnatal development. (D) Maturation and elimination of the uncrossed ventrolateral CST is impaired in *Emx1::Cre:Plexa1* ^*floxf/flox*^ mice, *Bax/Bak* mutants or when the activity of the motor cortex is silenced. EI: excitatory interneuron; InI: inhibitory interneuron; MN: motoneuron. Orange/red: segregated CST circuits innervating antagonistic muscles. Green: a single CST circuit innervates both flexor (light green) and extensor (light blue) muscles. Dark blue: normal anatomy of the CST. Red: aberrant maintenance of the uncrossed ventrolateral CST. [Color figure can be viewed at wileyonlinelibrary.com]

The maturation of the CST projection pattern is mainly an activity‐dependent process.^66^ CST circuits controlling forelimb antagonistic muscles are refined during postnatal development in mice.^67^ Antagonistic forelimb muscles are controlled by segregated CST circuits at early postnatal stages. By contrast, in adult mice, the proportion of antagonistic muscle pairs that are innervated by a single CST circuit through excitatory and inhibitory interneurons, respectively, increases (Fig. 4A). Intracortical microstimulation of the motor cortex elicits coactivation of antagonistic muscles in juvenile mice, but not in adults. This suggests that in adult mice, a single CST population controls the harmonious contraction–relaxation of antagonistic muscle pairs. The non‐apoptotic BAX/BAK signaling pathway is involved in neural circuit formation and maturation. In *Bax/Bak* double mutant mice, the maturation of the CST is impaired: antagonistic muscles remain innervated by segregated CST circuits in adulthood (Fig. 4B). This is associated with aberrant co‐activation of antagonistic muscles upon stimulation of the motor cortex in adult mice and impaired motor performance during skilled reaching tasks. However, the results of cortical stimulation experiments in mice should be interpreted with caution. Direct stimulation of the CST in mice has only limited effects on motoneurons.^5^ This suggests that the effects of cortical stimulation in mice may result from the activation of other descending pathways, such as the reticulospinal tract.

Ipsilateral ventrolateral CST projections are normally eliminated during postnatal development (Fig. 4C). This is not the case in the absence of the plexin‐A1 receptor (Fig. 4D),^52^ raising the possibility that plexin‐A1/semaphorin‐6D and BAX/BAK signaling pathways interact in the refinement of ipsilateral CST projections to the spinal cord.^68^ In *Bax/Bak* double mutants, the ipsilateral ventrolateral CST aberrantly persists in adult mice. Inhibition of CST activity during postnatal development leads to similar consequences (Fig. 4D), with abnormal bilateral muscle responses following unilateral cortical stimulation. The activity‐dependent BAX/BAK signaling pathway is thus required for the refinement of ipsilateral CST projections. Interestingly, *Plexa1* expression was decreased in *Bax/Bak* double mutants or when CST activity was inhibited.^68^ This suggests that PLEXA1 acts as a downstream effector of the activity‐dependent BAX/BAK signaling pathway to drive the elimination of ipsilateral CST projections during postnatal development.

### Concluding Remarks

Although there are important interspecies differences in CST anatomy, the pyramidal decussation is conserved in most mammals. The fitness advantage conferred by the crossed anatomy of the CST is probably linked to the crossed anatomy of visual pathways, allowing optimal escape behavior when facing a threatening stimulus^69^ (Panel 4). Interestingly, the role of DCC and netrin‐1 in CST midline crossing is also common to all mammals: mutations of either of these genes results in abnormal pyramidal decussation and MMs in both mice and humans.

The CST projection pattern along the rostrocaudal axis of the CNS underwent a major shift during mammalian evolution. In rodents, the CST exhibits broad connections with sensory systems, in particular at supraspinal levels. By contrast, CST projections are much more focused on the spinal motor system in non‐human primates. However, the main evolutionary divergence concerns the CST projection pattern in the spinal cord grey matter. The location of CST projections switched from a dorsal to a ventral position across mammalian evolution. For a long time it was thought that this anatomical switch paralleled the appearance of direct cortico‐motoneuronal connections in higher primates, with a subsequent enhancement of manual dexterity. This view was challenged by the recent description of transient direct cortico‐motoneuronal connections in rodents, which are eliminated during postnatal development. Genetic manipulations resulting in the maintenance of direct cortico‐motoneuronal connections in adult rodents are associated with increased manual dexterity. Paradoxically, this reveals that higher dexterity might not confer any fitness advantage to rodents.

Several important questions remain unanswered. (1) The CST makes broad connections with non‐motor structures in rodents, while it is more focused on spinal motor system in macaques. What is the function of these connections with sensory systems in rodents? Why were they not conserved across mammalian evolution? (2) The genetic determinants underlying the specification of distinct CST populations targeting the cervical and thoracolumbar spinal cord have been elucidated. How does this translate into the functional specialization of the forelimbs and hindlimbs in rodents? Are these genetic determinants conserved in humans? (3) What is the evolutionary tradeoff explaining the elimination of direct cortico‐motoneuronal connections in rodents?

Overall, this evolutionary prospect will be critical for identifying the molecular mechanisms controlling human CST development and the formation of direct cortico‐motoneuronal connections. The identified genes and proteins could translate into potential therapeutic targets for brain or spinal cord regeneration after injury.^40^

#### Panel 1: Mirror Movements

Mirror movements (MMs) are involuntary movements on one side of the body that mirror voluntary movements on the other side and constitute the main neurological manifestation in human congenital mirror movement disorder (CMM). CMM is a rare genetic disorder transmitted in an autosomal dominant manner in which MMs are the main clinical abnormality. MMs predominate in the distal upper limbs, leaving affected individuals unable to perform independent actions with two hands or to perform purely unimanual movements. The main culprit genes are *RAD51*, *DCC*, and *NTN1*.^8^, ^9^, ^10^ While the role of *DCC* and *NTN1* in axonal guidance has long been known, the mechanisms underlying MMs in *RAD51* patients remain unknown.^70^ Two additional genes have been associated with CMM disorder in unique families: *DNAL4* (with recessive transmission) and *ARHGEF7*.^11^, ^13^ MMs can also be a minor feature of complex developmental disorders, such as Kallmann syndrome and Klippel–Feil syndrome.^1^, ^71^ In healthy humans, during unimanual movement, the active motor cortex (contralateral to the intended movement) inhibits the ipsilateral motor cortex via fibers that pass through the corpus callosum, thereby restricting the motor output to the active primary motor cortex. This inhibition of one motor cortex by the other is called interhemispheric inhibition (IHI). The motor command is then transmitted unilaterally to the contralateral spinal cord via the crossed CST. Two abnormalities have been consistently associated with MMs^3^: (1) decreased IHI during movement preparation and execution, resulting in bilateral primary motor cortex activation during intended unimanual movement and (2) abnormal guidance of the CST at the midline resulting in bilateral, instead of mostly contralateral, transmission of the motor command to the spinal cord.

#### Panel 2: Transcriptional Analyses for CST Development

Identifying the genetic determinants responsible for CST neurons specification and the targeting of specific spinal segments is a two‐steps process.^37^, ^40^, ^41^ The first step is to isolate a specific CST neurons subpopulation. This can be achieved by injecting a retrograde tracer at a specific spinal level, followed by microdissection of the cortex. CST neurons are then purified using fluorescence‐activated cell sorting (FACS), enabling the separation of labeled CST neurons from other, unlabeled, cell types. The second step is to identify genes that are selectively expressed in the isolated CST population. This requires transcriptional analysis, meaning a sequencing of the RNAs present within these neurons. Transcriptional analysis can be performed either on a population of CST neurons^37^ or on single cell.^40^, ^41^ Among the great number of transcripts that are identified, statistical analyses are required to select a smaller number of candidate genes that are differentially expressed between specific CST subpopulations.

#### Panel 3: Intersectional Viral Tools to Map CST Projections

The visualization of axonal tracts in animal models relies on the utilization of tracers. Tracers can be molecules or viral particles that can enter the neuron and then be visualized via a chemical reaction or the expression of a fluorescent protein. However, anterograde or retrograde tracers alone are insufficient to selectively label the CST. Indeed, injection of an anterograde tracer in the primary motor cortex will label not only the CST but also other descending projections such as corticothalamic or corticobulbar tracts. Conversely, injection of a retrograde tracer into the spinal cord labels a large number of spinal‐projecting circuits, not only the CST. To overcome this limitation, several recent studies used an elegant intersectional viral approach in rodents and non‐human primates.^37^, ^44^, ^45^ Sinopoulou et al. first injected a viral construct expressing the Cre recombinase in the caudal cervical spinal cord (C7‐T1) of adult rats and rhesus macaques, which was retrogradely transported to CST neurons in the cortex.^45^ They then injected various viral vectors expressing Cre‐dependent tracers into the primary motor cortex. As a result, the tracers were specifically expressed in the CST neurons projecting to the caudal cervical spinal cord, in contrast to other descending tracts of the primary motor cortex that did not express the Cre.

#### Panel 4: Why Is the CST Crossed?

The anatomy of the CST is strikingly conserved from the cortex to the pyramidal decussation across mammals, with only rare cases of uncrossed CST.^1^ By contrast, there are important interspecies differences at the spinal level. This suggests that the crossed anatomy of the CST confers an evolutionary advantage, a hypothesis remaining unchallenged since it was formulated by Santiago Ramón y Cajal over a 100 years ago.^69^, ^72^ He speculated that the crossed anatomy of the CST was constrained by the anatomy of the visual system. The images formed on the retina are inverted by the ocular lens, and Ramón y Cajal proposed that the decussation of visual pathways at the optic chiasma is required to restore the continuity of the visual scene in the brain. As a result, the left visual field projects to the right hemisphere and vice versa. Ramón y Cajal further suggested that the crossed anatomy of tactile and motor tracts such as the CST is a consequence of the crossed anatomy of the visual system. It would allow the convergence of a broad range of sensory and motor modalities within the same hemisphere, thus optimizing the interaction with the environment. Escape behaviors nicely illustrate this hypothesis.^69^ When a threatening stimulus is present in the left visual field, it activates the right hemisphere of the brain and thus generates a movement of the left limbs through the crossed CST. This generates a movement to the right side, in the opposite direction to the threat.

## Author Roles

(1) Research Project: A. Conception, B. Organization, C. Execution; (2) Statistical Analysis: A. Design, B. Execution, C. Review and Critique; (3) Manuscript Preparation: A. Writing of the First Draft, B. Review and Critique.

E.R.: 3A, 3B.

C.D.: 3B.

Q.W.: 3A, 3B.

## Financial Disclosures

C.D. and Q.W. have no disclosures to declare. E.R. received honoraria for speaking from Orkyn, Aguettant, and Elivie, and for participating on an advisory board from Merz‐Pharma. He received research support from Merz‐Pharma, Orkyn, Aguettant, Elivie, Ipsen, Everpharma, Enjoysharing, Fondation Desmarest, AMADYS, ADCY5.org, Fonds de dotation Patrick Brou de Laurière, Agence Nationale de la Recherche, Societé Française de Médecine Esthétique, and the Dystonia Medical Research Foundation.

## Data Availability

Data sharing not applicable to this article as no datasets were generated or analysed during the current study.
